# High MMP-11 expression associated with low CD8+ T cells decreases the survival rate in patients with breast cancer

**DOI:** 10.1371/journal.pone.0252052

**Published:** 2021-05-26

**Authors:** Hyung Suk Kim, Min Gyu Kim, Kyueng-Whan Min, Un Suk Jung, Dong-Hoon Kim

**Affiliations:** 1 Division of Breast Surgery, Department of Surgery, Hanyang University Guri Hospital, Hanyang University College of Medicine, Guri, Gyeonggi-do, Republic of Korea; 2 Department of Surgery, Hanyang University Guri Hospital, Hanyang University College of Medicine, Guri, Gyeonggi-do, Republic of Korea; 3 Department of Pathology, Hanyang University Guri Hospital, Hanyang University College of Medicine, Guri, Gyeonggi-do, Republic of Korea; 4 Department of Obstetrics and Gynecology, Hanyang University Guri Hospital, Hanyang University College of Medicine, Guri, Gyeonggi-do, Republic of Korea; 5 Department of Pathology, Kangbuk Samsung Hospital, Sungkyunkwan University School of Medicine, Seoul, Republic of Korea; Fondazione IRCCS Istituto Nazionale dei Tumori, ITALY

## Abstract

Matrix metalloproteinase-11 (MMP-11) promote cancer invasion and metastasis through degrading the extracellular matrix. Protein degradation by MMP-11 in tumor cells may progressively suppress cancer surveillance activities with blocking immune response in breast cancer. The aim of study is to analyze clinicopathological parameters, molecular interactions and anticancer immune response in patients with MMP-11 expression and to provide candidate target drugs. We investigated the clinicopathologic parameters, specific gene sets, tumor antigenicity, and immunologic relevance according to MMP-11 expression in 226 and 776 breast cancer patients from the Hanyang University Guri Hospital (HUGH) cohort and The Cancer Genome Atlas (TCGA) data, respectively. We analyzed pathway networks and in vitro drug response. High MMP-11 expression was associated with worse survival rate in breast cancer from HUGH cohort and TCGA data (all *p* < 0.05). In analysis of immunologic gene sets, high MMP-11 expression was related to low immune response such as CD8+T cell, CD4+T cell and B cell. In silico cytometry, there was a decrease of cancer testis antigen and low tumor infiltrating lymphocyte in patient with high MMP-11 expression: activated dendritic cell, CD8+T cell, CD4+ memory T cell, and memory B cell. In pathway networks, MMP-11 was linked to the pathways including low immune response, response to growth hormone and catabolic process. We found that pictilisib and AZ960 effectively inhibited the breast cancer cell lines with high MMP-11 expression. Strategies making use of MMP-11-related hub genes could contribute to better clinical management/research for patients with breast cancer.

## Introduction

Breast cancer is the most common cancer diagnosed in women and represents a heterogeneous group of diseases with many clinicopathological characteristics. Although the diagnosis and treatment of breast cancer continues to be developed, it remains the leading cause of cancer-related death in women worldwide [1]. Identifying the prognostic biomarkers of breast cancer is an important aspect of determining the treatment of breast cancer and predicting the response to treatment. Recently, tumor biological characteristics and molecular genetic characteristics have been considered important prognostic biomarkers because of the development of molecular techniques [2]. Therefore, it is essential to identify novel therapeutic and prognostic biomarkers at the molecular level to improve the management of breast cancer patients, which will also provide a better understanding of breast cancer.

The tumor microenvironment, including the extracellular matrix, is a complex system, and the immune cells in the tumor microenvironment are known to be important factors influencing cancer progression [3]. Studies of the tumor microenvironment have confirmed that stromal cells and the host’s immune system play an important role in determining the malignant phenotype [4]. Matrix metalloproteinases (MMPs), which comprise a large family of zinc-dependent endopeptidases, can degrade the constituents of the extracellular matrix (ECM), which is the most important physiological barrier for the movement of tumor cells, and thereby promote the invasion and metastasis of cancer cells [5–7]. Previous studies have described the existence of MMPs in several types of cancers, including breast cancer, and the important role of MMPs in the progression of cancer and metastasis by remodeling the tumor microenvironment [6–8].

MMP-11, which is also named stromelysin-3, was first shown to be highly expressed in stromal cells in invasive breast cancer [9]. Most MMPs are secreted in the form of inactive proenzymes, while MMP-11 is processed in the cytoplasm and secreted in active enzyme form [10]. The special ability of MMP-11, which is different from other MMP members, suggests that it plays a distinct role in tumor development and progression. Previous studies have shown that overexpression of MMP-11 is associated with a large tumor size, lymph node involvement, high histologic grade, estrogen receptor (ER), HER2, and poor survival in breast cancers [11, 12]. Overexpression of MMP-11 plays an important role as a potential predictor for early diagnosis, prognosis prediction and adaptive treatment. Immunological targeting of MMPs has been proposed in several studies, such as MMP2 and MMP7 being proposed as a candidate for antigen-specific immunotherapy [13, 14]. Recent studies have suggested that MMP-11 may be a good candidate target for cancer immunotherapeutic treatment [15]. However, a correlation between the clinical outcomes of breast cancer and high MMP-11 expression has been reported, but little has been reported about the anti-tumor immunity role of MMP-11 expression in breast cancer.

The present study aimed to assess whether MMP-11 is related to clinicopathological parameters and the survival of breast cancer patients based on data from patients seen at Hanyang University Guri Hospital (HUGH) and from the The Cancer Genome Atlas (TCGA) database [16]. To evaluate the relationship between the high expression of MMP11 and the immune response in breast cancer patients, we focused on evaluating the high expression of MMP-11 associated immune gene sets and genes, the different types of involved immune cells and network-based pathway. Also, we performed in vitro drug screening tests in breast cancer cell lines according to MMP-11 expression to identify suitable target therapy.

## Methods

### Patient selection

This study included 226 patients with invasive ductal carcinoma (IDC) of the breast who were to undergo surgery at HUGH in Korea between 2005 and 2015. The Reporting Recommendations for Tumor Marker Prognostic Studies (REMARK) criteria were followed throughout this study [17]. The inclusion criteria were as follows: 1) patients with invasive ductal carcinoma of the breast confirmed by pathologists with known medical records; and 2) patients who did not undergo neoadjuvant chemotherapy. Patients with missing paraffin blocks of tumor tissues or incomplete clinical outcome data were excluded. We assessed the T and N stage, histopathological grade/differentiation and lymphovascular and perineural invasion (**S1 Table**).

### Ethics approval

This study protocol was approved by the Institutional Review Board of Hanyang University Guri Hospital (IRB number: 2020–02–012) and was performed according to the ethical standards of the Declaration of Helsinki, as revised in 2008. The review conducted by our institutional review board confirmed that informed consent was not necessary for this study.

### Tissue microarray construction and immunohistochemistry

The tissue microarray (TMA) blocks were assembled using a tissue array instrument (AccuMax Array; ISU ABXIS Co., Ltd., Seoul, Korea). We used duplicate 3 mm-diameter tissue cores (tumor components in a tissue core > 70%) from each donor block. Four-micrometer sections were cut from the TMA blocks using routine techniques. Immunostaining for MMP-11 (1:200, Lab Vision Corporation, USA), ER (1:200, Lab Vision Corporation, Fremont, CA, USA), progesterone receptor (PR) (1:200, Dako, Glostrup, Denmark), human epidermal growth factor receptor 2 (HER2) (1:1, Ventana Medical Systems Tucson, Arizona, USA), P53 (1:5000, Cell Marque, Hot Spring, AR, USA) and Ki67 (1:200; MIB-1, Dako, Glostrup, Denmark), anti-CD8 (clone 4B11 Leica Biosystems, Newcastle, UK) and anti-CD4 (clone 4B12 Leica Biosystems, Newcastle, UK) were performed using the Bond Polymer Refine Detection System (Leica Biosystems Newcastle Ltd. Newcastle, UK) according to the manufacturer’s instructions.

**Fig 1:**
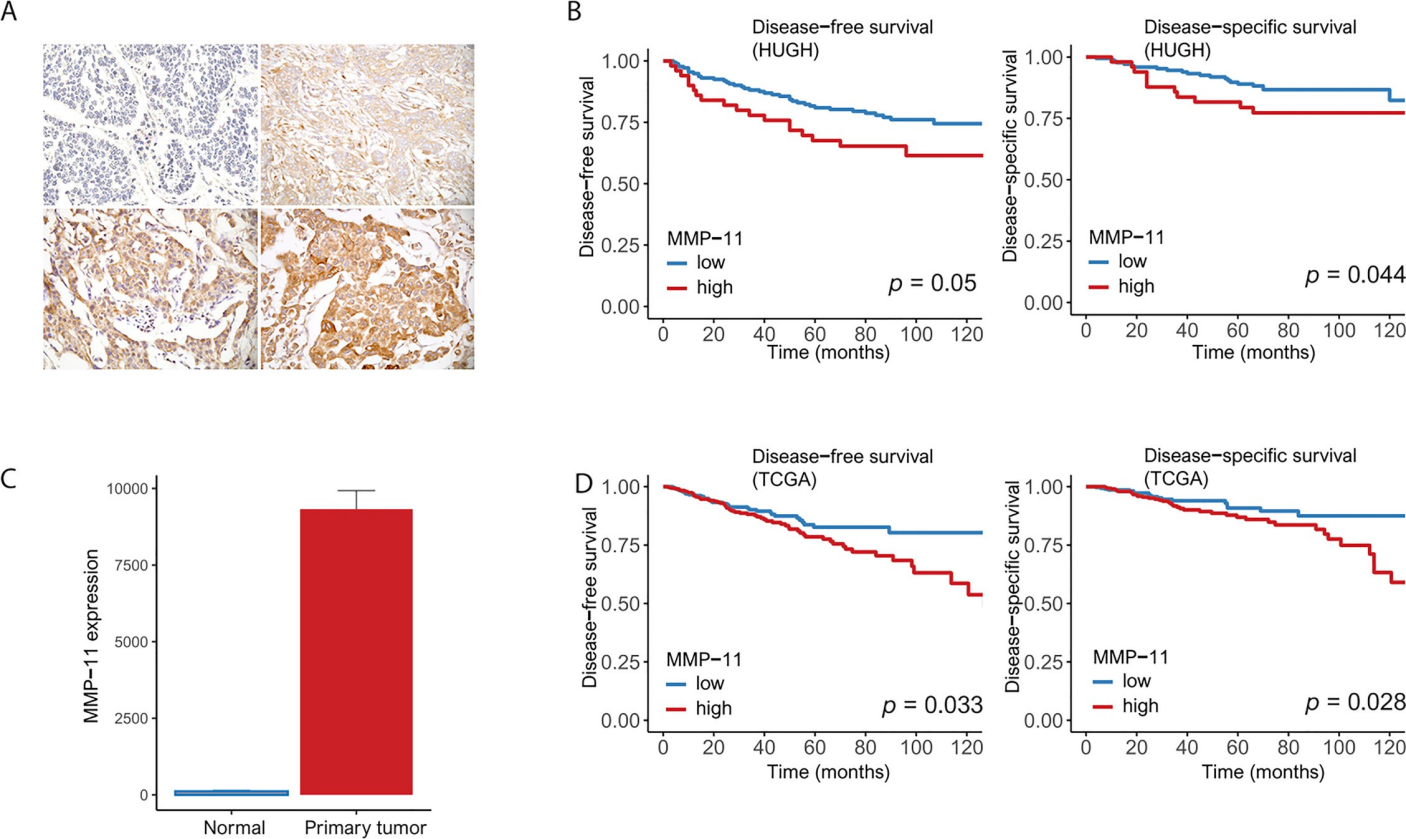
(A) Representative microphotographs revealing (left top) negative, (right top) weak, (left bottom) moderate and (right bottom) strong intensity MMP-11 expression using immunohistochemical staining (original magnification × 400). (B) HUGH cohort: High MMP-11 expression was associated with poor disease-free survival (left) and disease-specific survival (right) in 226 patients (*p* = 0.05 and 0.044, respectively). (C) TCGA: High MMP-11 expression in primary tumors compared to that in normal tissues. (D) TCGA: High MMP-11 expression was associated with poor disease-free survival (left) and disease-specific survival (right) in 776 patients in TCGA (*p* = 0.033 and 0.028, respectively).

MMP-11 cytoplasmic staining intensity in tumor cells was scored on a scale of 0 to 3 (0 = negative; 1 = weak; 2 = moderate; 3 = strong). The percentage of MMP-11 positive tumor cells also was scored into 1 of 4 categories: 1 (0–25%), 2 (26–50%), 3 (51–75%), or 4 (76–100%). The level of MMP-11 staining was analyzed as an immunoreactive score (IRS), which was calculated by multiplying the scores of staining intensity and the percentage of positive cells. MMP-11 expression was determined as either low (IRS ≤ 6) or high (IRS > 6) [18] (**Fig 1A**). To determine the optimal cutoff values of MMP-11 in HUGH, receiver operating characteristic (ROC) curves plotting sensitivity versus 1 –specificity were used. The cutoff value calculated by the ROC was used to evaluate the relationship between cancer specific death events and MMP-11 expression. ROC exhibited good discriminatory power considering the death events for MMP-11 expression of the tumor cells (area under the ROC = 0.662) (**S1 Fig**).

### Gene sets, in silico cytometry and network analysis based on the TCGA database

We obtained 776 IDC cases with RNA-Seq data from TCGA database [16]. The RNA seq from TCGA was calculated. On basis of the cancer specific death events in the TCGA database, the values of MMP-11 were divided into low and high using the most sensitive and specific value in ROC curve analysis. The cutoff value was 5114.265 (log2-transformed score 12.32059). MMP-11 expression was determined as either low (log2 -transformed scores < 12.32059) or high (log2 -transformed scores > 12.32059) based on the median value. Gene Set Enrichment Analysis (GSEA) is a method of analyzing and interpreting microarray and other such data based on biological information. These biological sets can be published information on a biochemical pathway or co-expression obtained in a previous experiment. We analyzed the significant gene sets using GSEA software (version 4.03) from the Broad Institute at MIT [19]. The gene set (4872 immunologic signatures) was used to identify the gene sets associated with high MMP-11 expression. For this analysis, 1,000 permutations were used to calculate the *p*-values, and the permutation parameters were set to a phenotype of *p* < 0.05, a false discovery rate (FDR) of < 0.2 and a family wise-error rate (FWER) of ≤ 0.4. The GSEA results can determine whether there is a relationship between immune cell-related gene sets and high MMP-11 expression. We applied in silico cytometry known as CIBERSORT to analyze the proportions of 22 subsets of immune cells using 547 genes [20]. For grouping of networks based on functionally enriched gene ontology (GO) terms and pathways, the pathway network analyses were visualized using Cytoscape software (version 3.8.0). To interpret the immunologic relevance of MMP-11 and its relevant elements in IDC, we performed functional enrichment analysis. We selected 300 top-ranked genes associated with high MMP-11 expression. We observed the closest relationship between various genes and high MMP-11 expression using kappa value based on six ontologies/pathway such as biological process, cellular component, immune system process, KEGG, pathways, and Wikipathways and clarify the functionally grouped gene ontology and pathway annotation networks using ClueGO application (version 2.5.6), an app for gene ontology analysis.) [21, 22].

### Data extraction from the GDSC database

We analyzed relationship between anticancer drug sensitivity and MMP-11 expression based on the Genomics of Drug Sensitivity in Cancer (GDSC, version 2) dataset, which contains data on the drug responses of approximately 50 breast cancer cell lines to 172 anticancer drugs [23]. In breast cancer cell lines with high MMP-11 expression (19 cell lines > 0 expression based on z-scores: CAL-148, CAL-51, UACC-893, MDA-MB-361, DU-4475, HCC2157, EFM-19, BT-549, HCC1569, BT-483, HDQ-P1, COLO-824, BT-474, HCC202, HCC1395, Hs-578-T, HCC1599, UACC-812, and T47D) or low expression (31 cell lines < 0: MFM-223, MCF7, HCC70, MDA-MB-231, MDA-MB-436, CAMA-1, HCC2218, MRK-nu-1, HCC1143, HCC38, MDA-MB-415, MDA-MB-453, JIMT-1, HCC1937, AU565, CAL-85-1, MDA-MB-157, ZR-75-30, MDA-MB-330, HCC1419, HCC1954, HCC1187, CAL-120, EFM-192A, HCC1806, HCC1428, BT-20, HCC1500, EVSA-T, OCUB-M, and MDA-MB-468), the drug response was defined as the natural log of the half-maximal inhibitory concentration (LN IC50). We found a drug sensitive when the amount of drug suppressing cells with high MMP-11 expression was lower than what was required to suppress cells with low MMP-11 expression. A drug was identified as an effective drug when the calculated LN IC50 value was decreased in cell lines with high MMP-11 expression and increased in those with low MMP-11 expression, i.e., when an inverse correlation was observed. Pearson’s correlation and Student’s t-test were used to assess the comparisons between the LN IC50 values and MMP-11 expression [24, 25].

### Statistical analysis

Correlations between clinicopathological parameters and MMP-11 expression were analyzed using the χ ^2^ test. Student’s t-test and Pearson’s correlation were used to examine the differences among continuous variables. Relapse-free survival (RFS) was defined as survival from the date of diagnosis to local recurrence. Disease-free survival (DFS) was defined as survival from the date of diagnosis to recurrence/new distant metastasis, with disease-specific survival (DSS) defined as survival from the date of diagnosis to cancer-related death. Overall survival (OS) time was defined as the time from the date of diagnosis to all-cause death. Survival rates were compared using the log-rank test and Cox regression analyses. A two-tailed *p*-value of <0.05 was considered statistically significant. All data were analyzed using R software packages and SPSS statistics (version 25.0, SPSS Inc., Chicago, IL, USA).

## Result

### Clinical manifestations of MMP-11

The detailed clinicopathological characteristics of the 226 patients in our cohort (HUGH) according to MMP-11 expression are described in **Table 1**. High MMP-11 expression was associated with negative ER and PR status, the presence of HER2, and high p53 expression (*p* = 0.046, 0.016, 0.005, and 0.01, respectively) (**Table 1**). High MMP-11 expression was related to DFS, DSS and RFS (*p* = 0.05, 0.044 and 0.035, respectively). There was no OS difference between low and high MMP-11 expression (*p* = 0.62). After adjustment for T and N stage, histological grade lymphovascular invasion and ER, significant correlations between high MMP-11 expression and low DFS or DSS remained (*p* = 0.041 and 0.049, respectively) (**Table 2**) (**Fig 1B**) (**S2 Fig**). In the breast cancer data from the TCGA database, MMP-11 was more highly expressed in primary tumors than in normal tissues (**Fig 1C**). There was a relationship between high MMP-11 expression and low DFS or DSS (*p* = 0.033 and 0.028, respectively) (**Fig 1D**).

**Table 1:** Clinicopathological parameters of MMP-11 in patient with invasive ductal carcinoma of the breast from HUGH cohort.

Parameter	MMP-11 (our cohort)		*p* value
	Low (n = 176), n (%)	High (n = 50), n (%)	
Age	48.0 ± 9.7	49.3 ± 11.3	0.445^2^
T classification			
1	71 (40.3)	14 (28.0)	0.112^3^
2	92 (52.3)	33 (66.0)	
3	13 (7.4)	3 (6.0)	
N classification			
0	80 (45.5)	21 (42.0)	0.464^3^
1	52 (29.5)	19 (38.0)	
2	20 (11.4)	6 (12.0)	
3	24 (13.6)	4 (8.0)	
Histological grade			
1	28 (15.9)	4 (8.0)	0.19^1^
2	85 (48.3)	23 (46.0)	
3	63 (35.8)	23 (46.0)	
Lymphatic invasion			
Negative	84 (47.7)	26 (52.0)	0.709^1^
Positive	92 (52.3)	24 (48.0)	
Perineural invasion			
Negative	148 (84.1)	43 (86.0)	0.914^1^
Positive	28 (15.9)	7 (14.0)	
Tumor necrosis			
Absence	102 (58.0)	30 (60.0)	0.923^1^
Presence	74 (42.0)	20 (40.0)	
ER			
Negative	46 (26.1)	21 (42.0)	0.046^1^
Positive	130 (73.9)	29 (58.0)	
PR			
Negative	63 (35.8)	28 (56.0)	0.016^1^
Positive	113 (64.2)	22 (44.0)	
HER2			
Negative	136 (77.3)	28 (56.0)	0.005^1^
Positive	40 (22.7)	22 (44.0)	
P53 percentage	23.7 ± 34.6	38.8 ± 40.5	0.01^2^
Ki-67 index	3.9 ± 9.5	2.8 ± 6.9	0.377^2^

T or N classification, 8^th^ edition; ER, estrogen receptor; PR, progesterone receptor; HER2, human epidermal growth factor receptor 2; MMP, matrix metalloproteinase

^1^Chi-square test

^2^Student’s t-test

**Table 2:** Disease-free and disease-specific survival analyses according to MMP-11 in 226 breast cancer patients (HUGH cohort).

Disease-free survival	Univariate^1^	Multivariate^2^	HR	95% CI	
MMP-11 (low vs. high)	0.05	0.041	1.846	1.025	3.324
Tumor stage (1,2 vs. 3,4)	<0.001	0.016	2.635	1.202	5.774
Nodal stage (0,1,2 vs. 3)	<0.001	0.001	3.013	1.586	5.725
Histological grade (1,2 vs. 3)	<0.001	0.240	1.486	0.767	2.877
Lymphovascular invasion (absence vs. presence)	<0.001	0.078	1.781	0.937	3.386
ER (negative vs. positive)	0.009	0.354	0.745	0.399	1.389
Disease-specific survival	Univariate^1^	Multivariate^2^	HR	95% CI	
MMP-11 (low vs. high)	0.044	0.049	2.048	1.004	4.180
Tumor stage (1,2 vs. 3,4)	<0.001	0.037	2.879	1.065	7.781
Nodal stage (0,1,2 vs. 3)	<0.001	0.004	3.382	1.477	7.745
Histological grade (1,2 vs. 3)	<0.001	0.442	1.410	0.588	3.383
Lymphovascular invasion (absence vs. presence)	<0.001	0.670	1.194	0.528	2.697
ER (negative vs. positive)	0.002	0.114	0.526	0.237	1.168

MMP, matrix metalloproteinase; ER, estrogen receptor

^1^Log rank test

^2^Cox proportional hazard model

### Gene set enrichment analysis, immune cell fraction and pathway network analyses in patients with high MMP-11 expression

**Fig 2:**
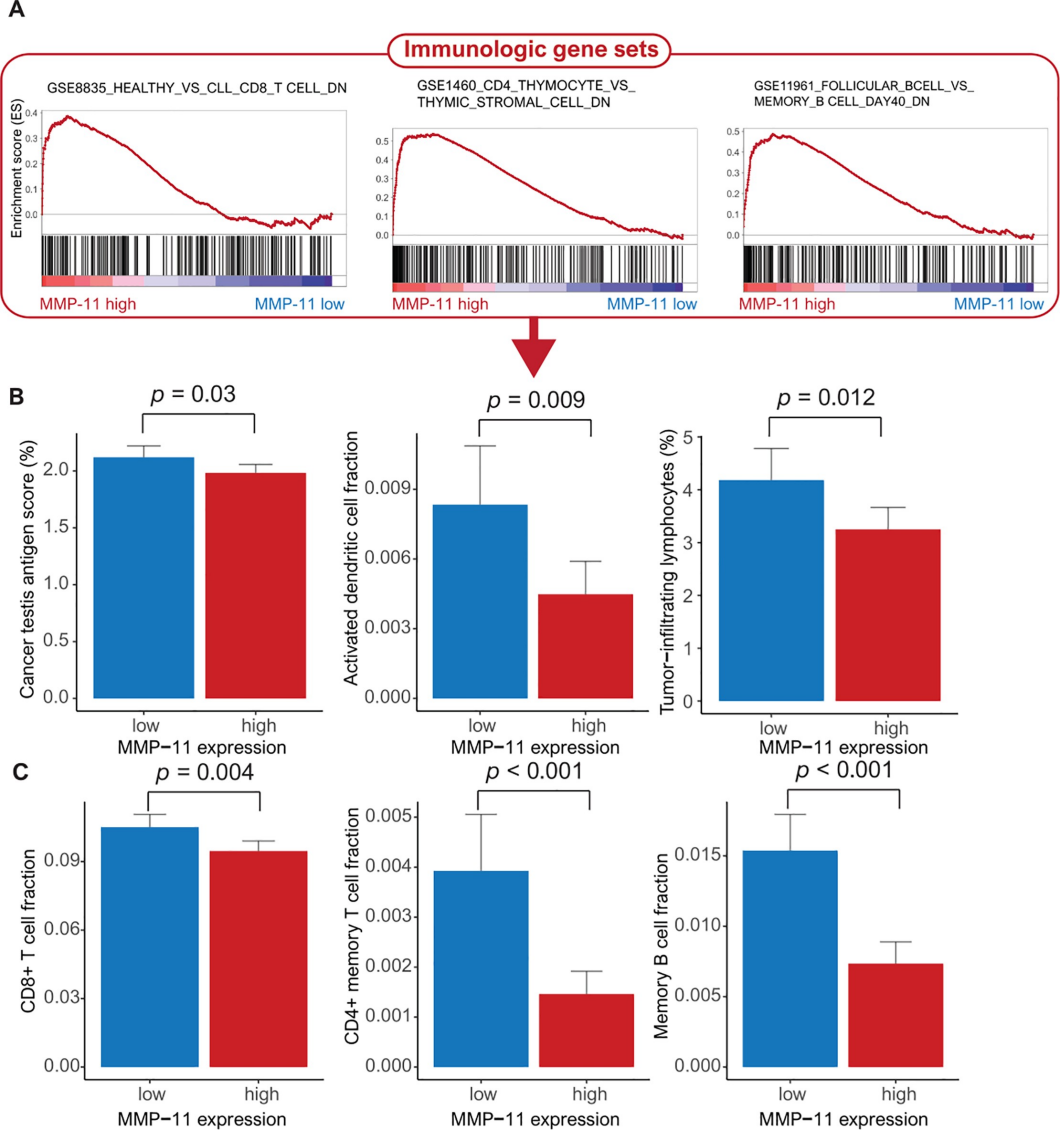
TCGA data (A) Gene set enrichment analysis of three MMP-11-dependent immunologic gene sets reveals the downregulation of CD8+ T cells, CD4+ T cells and B cells. (B) Bar plots of MMP-11 expression and the following parameters: (B) cancer testis antigen, activated dendritic cells, tumor-infiltrating lymphocytes, (C) CD8+ T cells, CD4+ memory T cells, and memory B cells.

**Fig 3:**
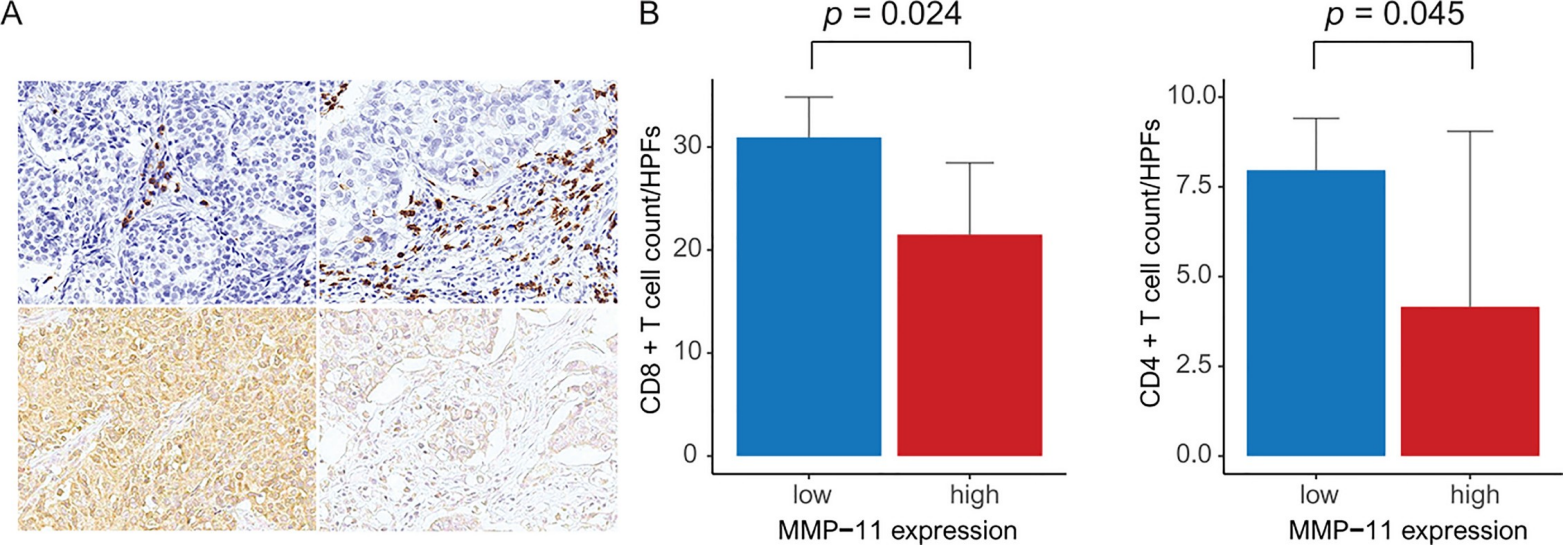
HUGH cohort (A) Representative microphotographs showing tumor-infiltrating CD8 T cells (brown): decreased CD8+ T cells (left top) in high MMP-11 expression (left bottom) and increased CD8+ T cells (right top) in low MMP-11 expression (right bottom) (original magnification × 400). (B) Bar plot of CD8 T cells (left) and CD4 T cells (right) per high-power field (× 400) (*p* = 0.024 and 0.045).

By using the TCGA data, we conducted GSEA to identify the gene sets associated with high MMP-11 expression. We found three significantly enriched gene sets related to the negative regulation of immune cells. We found that high MMP-11 expression was associated with the downregulation of the gene sets linked to CD8+ T cells, CD4+ T cells and memory B cells, respectively (**Fig 2A**). We conducted in silico flow cytometry to evaluate the relationship between MMP11 expression and various immune cells. As a result, cancer testis antigen (CTA), activated dendritic cells (DC) and tumor-infiltrating lymphocytes (TILs) were decreased in patients with high MMP-11 expression compared to those with low MMP-11 expression (*p* = 0.03, 0.009 and 0.012, respectively) (**Fig 2B**). High MMP-11 expression was related to low levels of CD8+ T cells, CD4+ memory T cells and memory B cells (*p* = 0.004, < 0.001 and <0.001, respectively) (**Fig 2C**). In HUGH cohort, high MMP-11 expression was associated with decreased CD8+ T cells and CD4+ T cells (*p* = 0.024 and 0.045, respectively) (**Fig 3**)

**Fig 4:**
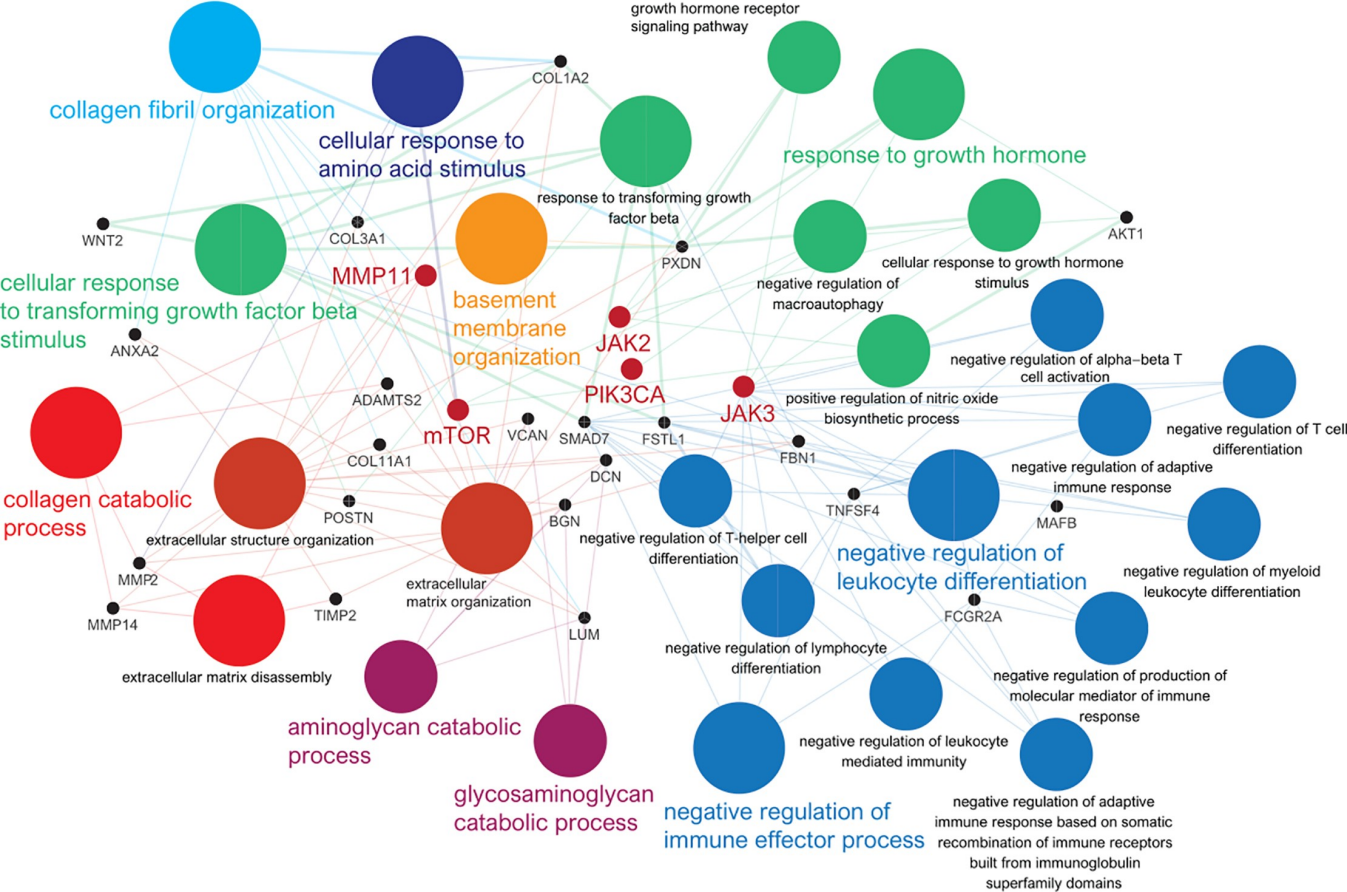
Grouping of networks based on functionally enriched GO terms and pathways using Cytoscape software (version 3.8.0) and ClueGO application (version 2.5.6) (https://cytoscape.org/): Functionally grouped networks are linked to their biological function, and only the most significant term of the group is labeled. MMP-11 is directly linked to collagen catabolic processes, collagen fibril organization and basement membrane organization, while it indirectly negatively regulates leukocyte differentiation and immune effector process. The specific hub genes directly or indirectly linked to MMP-11 included MTOR, PIK3CA, JAK2 and JAK3.

In the pathway network analysis based on GSEA, we found that high MMP-11 expression was directly linked to collagen catabolic processes, collagen fibril organization and basement membrane organization, and indirectly linked to negative regulation of leucocyte differentiation and immune effector process, cellular response to transforming growth factor beta stimulus, response to growth hormone, cellular response to amino acid stimulus, and aminoglycan catabolic process (**Fig 4**). We identified specific hub genes, such as mammalian target of rapamycin (mTOR), phosphatidylinositol-4,5-bisphosphate 3-kinase catalytic subunit α (PIK3CA), Janus kinase (JAK) 2 and JAK3, which are directly or indirectly linked to MMP-11, in a pathway-based network.

### Drug screening in breast cancer cell lines with high MMP-11 expression

**Fig 5:**
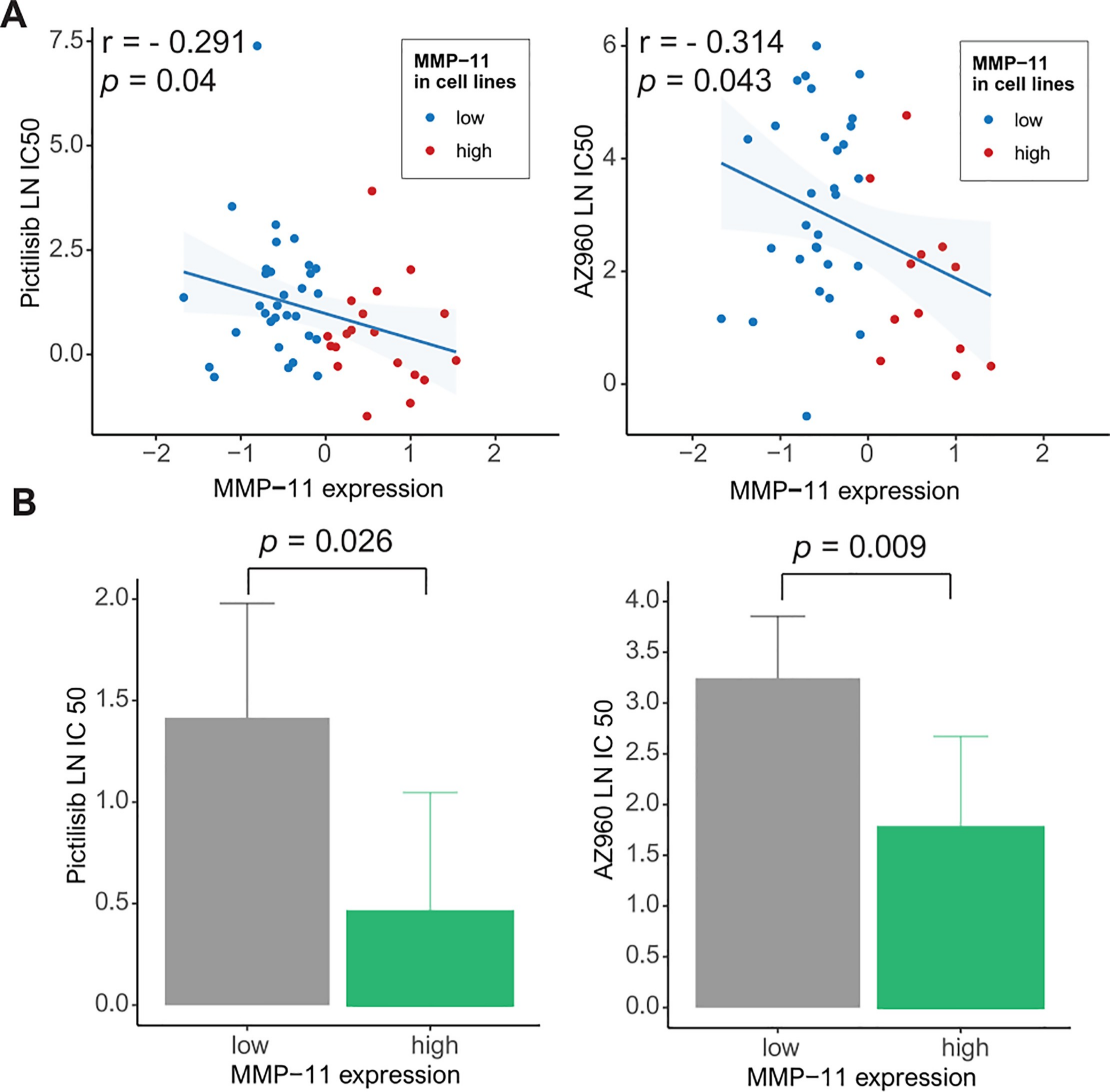
Genomics of Drug Sensitivity in Cancer (GDSC) database analysis: (A) Pearson’s correlation analysis showing the natural log of the half-maximal inhibitory concentration (LN IC50) values of pictilisib and AZ960 in breast cancer cells (blue, low MMP-11 expression; red, high MMP-11 expression) (left). Bar plot showing the LN IC50 values of pictilisib and AZ960 in breast cancer cell lines with low (gray) and high (green) MMP-11 expression (*p* = 0.026 and 0.009, respectively) (error bars: Standard errors of the mean) (right).

On the basis of the GDSC data, we analyzed drug sensitivity patterns in 50 breast cancer cell lines with high MMP-11 expression based on 172 drugs. Using Pearson’s correlation analysis, we considered drugs with a high negative correlation between MMP-11 expression and the LN IC50 value as effective MMP-11-targeting drugs. Pictilisib and AZ960 reduced the growth of cancer cell lines with high MMP-11 expression [pictilisib: r = -0.291, *p* = 0.04 (Pearson’s correlation) and 0.026 (Student’s t-test); AZ960: r = -0.314, *p* = 0.043 and 0.009] (**Fig 5A and 5B**).

## Discussion

This study revealed that high MMP-11 expression was correlated with worse clinicopathologic parameters, such as a high histologic grade, negative ER and PR status, and the presence of HER2. Our results showed that high MMP-11 expression was significantly related to poor DFS/DSS in our cohort (HUGH). Using TCGA database for validation of this results, we analyzed the survival rate according to high MMP-11 expression. High MMP-11 expression was associated with worse DFS/DSS in TCGA database. Also, our results revealed that MMP-11 expression was higher in primary cancer than normal breast tissue. We considered that MMP-11 could play an important role in breast cancer progression and metastasis and predicting clinical outcomes.

The tumor microenvironment can be affected by angiogenesis, reactive stromal fibroblasts, the immune response and proteinase production and can affect cancer progression [15]. MMPs, a family of zinc-dependent endopeptidases, can support stromal invasion of cancer cells via ECM modification [7]. One of the MMP members, MMP-11, called stromelysin-3, was first identified in breast cancer tissue. MMP-11 may enhance cancer development and progression by proteolytic degradation of the endothelial basement membrane [10]. Many studies have shown that high MMP-11 expression is associated with poor clinical outcomes in lung cancer, colorectal cancer, hepatocellular carcinoma, osteosarcoma, prostate cancer and breast cancer [26–31]. In our study of the HUGH cohort and TCGA database, high MMP-11 expression was associated with poor prognosis as well as a decline in DFS and DSS. Previous studies demonstrated that high MMP-11 was associated with poor prognosis in the breast cancer [32, 33].

The molecular mechanisms and pathways of carcinogenesis according to high MMP-11 expression in breast cancer have not yet been completely explained. In the GSEA, we identified that the specific gene sets associated with the downregulation of CD8+ T cells, CD4+ T cells and B cells, which play an important role in the complete elimination of cancer cells, were linked to high MMP-11 expression. Unexpectedly, Our study showed that high MMP-11 expression associated with a low antitumoral immune response (TILs, CD8+ T cells, CD4+ T cells, and memory B cells) and decreased the CTA score and activated DC fraction [34]. CTA is a category of tumor associated antigens. Immunogenic CTA can induce the expansion of CD8 + T cells that can reject tumor cells (cell immunity) or activate B cells that cause a humoral response in the form of tumor-specific antibodies [35]. A decreased immune reaction causes the difficulty of completely eliminating cancer cells, leading to cancer progression.

The stromal extracellular matrix influences anti-tumor immune system through controlling the positioning and migration of T lymphocytes [36]. A previous study demonstrated that modulation of C-X-C motif chemokine 11 (CXCL11) by MMPs might reduce the antitumor immune response and thus have direct consequences on tumor growth [37]. Therefore, tumorigenesis caused by high expression of MMP-11 did not result from increased cancer cell proliferation, but from decreased cancer cell death through apoptosis and necrosis [38]. Increased TIL is known to be a good prognostic factor for cancer because lymphocytes play an important role in anti-cancer immunity. Among lymphocytes, CD8+T cells play critical role in anti-cancer immunity. Our study showed that high MMP-11 expression was related to low levels of CD8+ T cells and CD4+ memory T cells. We hypothesize that the degradation of ECM by MMP-11 may have secondary effects on the immune system [39]. Moreover, we considered that the exposure of antigenicity by MMP-11 could be insufficient to recruit different antitumoral immune cells to the tumor site in breast cancer. Previous studies have shown that MMP-9 inhibition promotes anti-tumor immunity through trafficking of T cells to the tumor [40]. Experimental studies in vitro and in vivo may be necessary to resolve these questions about MMP-11. In network-based analyses, we showed that MMP-11 was linked to different genes, such as MTOR, JAK2, PIK3CA, and JAK3, and specific pathways, including those associated with catabolic processes and negative regulation of immune processes, suggesting an important association of breast cancer progression with high MMP-11 expression [41]. Previous studies have shown an association between MMP-11 and the hyperactivating the IGF-1-mediated PI3K/AKT signaling cascade [28]. Also, it has been shown that MMP expression is regulated by the signaling mechanisms of Interleukin 6 and Interleukin 8 through the JAK2/STAT3 pathway [42]. These results support our results.

The GDSC database, which includes data from drug screening in cancer cell lines, is available to investigate drug sensitivity [24]. We evaluated the sensitivity to pictilisib and AZ960 in breast cancer cell lines with high MMP-11 expression and those with low MMP-11 expression. Despite a weak correlation, Pictilisib reduced the growth of cancer cell lines with high MMP-11 expression. And, AZ960 had meaningful effect (r = -0.314) in breast cancer cell lines exhibiting high MMP-11 expression. Pictilisib is a potent and selective oral inhibitor of the class I PI3K pathway, which is one of the most commonly activated signaling pathways that regulates cell proliferation, survival and migration [43, 44]. In previous studies, pictilisib was shown to be a selective JAK3 inhibitor. High PIK3CA expression was associated with in vitro sensitivity of breast cancer cell lines to pictilisib [45, 46]. AZ960, a pyrazolo-nicotinonitrile analog, was reported to be a tight-binding ATP-competitive JAK2 inhibitor. The compound demonstrated greater than 3-fold selectivity for JAK2 over JAK3 when tested in biochemical assays [47]. The JAK2-STAT3 signaling pathway is a crucial activator of cell migration and cancer metastasis [48, 49]. We identified specific hub genes, such as mTOR, PIK3CA, JAK 2 and JAK3, which are directly or indirectly linked to MMP-11. Therefore, pictilisib and AZ960 may contribute to an improved treatment strategy for resistance to chemotherapy in breast cancer with high MMP-11 expression. Unlike the responses in breast cancer cell lines with high MMP-11 expression, the therapeutic responses in patients with breast cancer may be highly heterogeneous and affected by various microenvironments and immune components, which could have effects on clinical applications. Along with in vivo studies, pictilisib- and/or AZ960-based clinical trials in breast cancer patients with high MMP-11 expression are needed in the future.

This study has some limitations that should be acknowledged. First, this study was conducted as a retrospective study, and because of this, the analysis of MMP-11 did not show sustained relationships over time as did prospective studies. Second, the experimental analysis of the relationship between MMP-11 and immune cells was not performed, and further in vitro and/or in vivo studies are necessary. Third, the relationship between the expression of MMP-11 by the molecular subtype of breast cancer and prognosis is not analyzed.

In summary, this study demonstrated that high MMP-11 expression was significantly associated with poor DFS/DSS and correlated with the downregulation of gene sets linked to CD8+ T cells, CD4+ T cells and B cells according to GSEA. In silico cytometry showed that high MMP-11 expression was associated with low tumor antigenicity, reduced TILs, including CD8+ T cells, CD4+ memory T cells and memory B cells, and low activation of dendritic cells. We identified that Pictilisib and AZ960 affected breast cancer cell lines with high MMP-11 expression. These candidate drugs may be used for the treatment of patients with high MMP-11 expression and resistance to chemotherapy.

We believe that medical oncologists and researchers will be interested in the role of MMP-11 in promoting tumor invasion by ECM breakdown and that these results will contribute to designing future experimental studies.

## Supporting information

S1 Fig ROC curve. ROC curve for determination of the optimal cut-off value for MMP-11 expression according to patient survival rate in invasive ductal carcinoma of the breast (area under the ROC: 0.662 in tumor cells).
(PDF)

S2 Fig HUGH cohort: High MMP-11 expression was associated with poor disease-free and disease-specific survival in 226 patients (p = 0.035 and 0.62, respectively). (PDF)

S1 Table Clinicopathological parameters. (PDF)
