# Supplementary material for: High MMP-11 expression associated with low CD8+ T cells decreases the survival rate in patients with breast cancer
Source: PLoS One. 2021 May 26;16(5):e0252052. doi: 10.1371/journal.pone.0252052 (PMC8153507; doi:10.1371/journal.pone.0252052)
Supplement: S1 Fig — ROC curve for determination of the optimal cut-off value for MMP-11 expression according to patient survival rate in invasive ductal carcinoma of the breast (area under the ROC: 0.662 in tumor cells). (PDF) [file pone.0252052.s001.pdf]

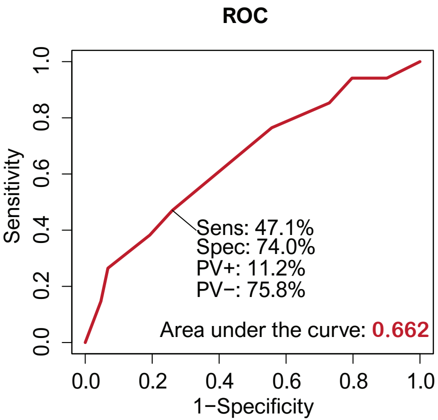

S1 Fig

ROC curve for determination of the optimal cut-off value for MMP-11 expression according to patient survival rate in invasive ductal carcinoma of the breast (Sens, sensitivity; Spec, specificity; PV+, positive predictive value and PV−, negative predicted value).
