# Supplementary material for: High MMP-11 expression associated with low CD8+ T cells decreases the survival rate in patients with breast cancer
Source: PLoS One. 2021 May 26;16(5):e0252052. doi: 10.1371/journal.pone.0252052 (PMC8153507; doi:10.1371/journal.pone.0252052)
Supplement: S2 Fig — (PDF) [file pone.0252052.s002.pdf]

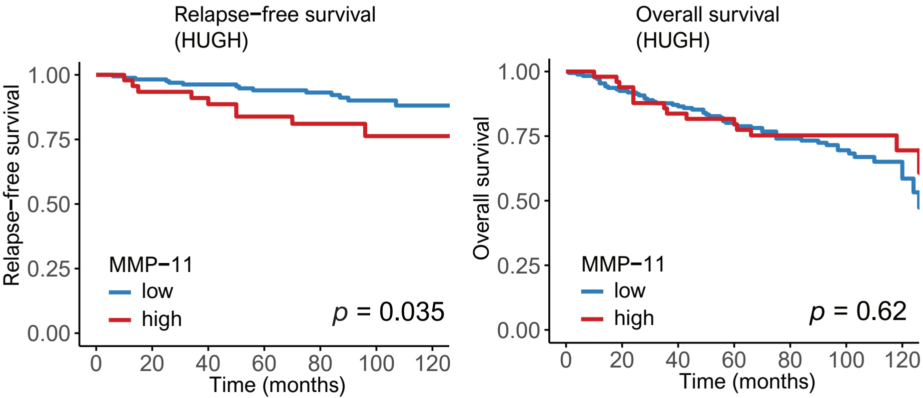

S2 Fig

HUGH cohort: High MMP-11 expression was associated with poor disease-free and disease-specific survival in 226 patients (*p* =0.035 and 0.62, respectively).
