# Supplementary material for: High MMP-11 expression associated with low CD8+ T cells decreases the survival rate in patients with breast cancer
Source: PLoS One. 2021 May 26;16(5):e0252052. doi: 10.1371/journal.pone.0252052 (PMC8153507; doi:10.1371/journal.pone.0252052)
Supplement: S1 Table — (PDF) [file pone.0252052.s003.pdf]

S1 Table. Clinicopathological parameters

Case	MMP11_intensity	MMP11_area (%)	CD4_count/HPF	CD8_count/HPF	DFS_times	DFS	DSS_times	DSS	ER	PR	HER2	P53(%)	Ki67(%)	T	N	Histological_Grade	Age	LVI	PNI	Necrosis
1	3	4	0	70	48	0	48	0	0	0	0	90	4	2	0	2	38	0	0	0
2	1	40	2	1	108	0	108	0	1	1	0	50	1	1	0	2	56	0	0	0
3	2	30	6	0	107	0	107	1	1	1	0	5	0	1	0	2	39	1	0	0
4	1	80	10	10	126	0	126	0	0	1	1	50	0	3	2	3	57	1	0	0
5	2	80	0	0	60	0	59	1	1	1	0	10	0	2	0	2	40	0	1	0
6	1	100	10	65	12	0	12	0	0	1	1	0	0	2	1	3	53	1	0	1
7	1	80	0	0	135	0	135	0	1	0	0	0	0	2	1	3	53	1	0	1
8	1	40	0	4	22	0	22	0	1	1	1	0	1	2	0	2	38	1	0	1
9	2	100	0	40	24	1	15	1	0	1	1	95	0	3	1	3	47	1	0	1
10	1	100	15	70	82	0	82	0	1	1	0	60	15	1	1	2	41	1	0	0
11	1	100	5	0	124	0	124	0	1	0	0	0	0	1	1	2	40	1	0	0
12	2	100	0	0	128	0	128	0	0	0	1	90	0	2	2	3	37	1	0	1
13	2	100	9	17	125	0	125	0	1	0	0	95	0	2	0	3	38	0	0	1
14	1	90	0	15	30	0	30	0	1	1	0	5	0	2	1	2	48	1	0	0
15	2	90	0	8	61	1	50	1	1	0	0	5	0	2	1	2	33	1	0	0
16	1	80	0	25	108	0	108	0	0	0	0	0	0	2	1	3	43	1	0	1
17	1	60	2	21	28	0	28	0	1	1	0	0	0	2	0	2	52	0	0	0
18	1	5	0	12	112	0	112	0	0	0	0	99	0	1	0	2	58	0	0	0
19	1	90	0	45	107	0	107	0	1	1	0	10	0	2	1	2	54	1	0	0
20	2	90	0	10	134	0	134	0	1	1	0	50	0	2	0	3	41	0	0	0
21	2	100	0	3	154	0	134	0	1	1	1	5	5	1	1	1	46	1	0	0
22	1	70	0	3	38	1	38	1	0	0	1	90	0	2	0	3	63	1	0	1
23	2	100	5	30	118	0	118	0	1	0	0	10	1	2	2	3	51	1	0	1
24	2	30	4	67	110	0	110	0	1	1	0	0	5	1	0	1	28	0	0	0
25	1	100	0	9	110	0	110	0	0	0	0	99	5	1	1	3	69	1	0	1
26	1	70	0	14	103	0	103	0	0	0	0	0	0	2	2	3	67	1	0	0
27	4	4	10	53	170	0	170	0	0	0	1	0	0	2	3	3	37	1	0	0
28	3	70	9	16	17	0	17	0	1	1	0	5	1	2	0	2	31	0	0	0
29	4	4	13	13	60	1	60	1	1	1	0	30	1	1	0	2	51	0	0	0
30	1	70	0	8	6	0	6	1	1	1	0	90	15	2	0	3	50	1	0	1
31	2	60	0	46	125	0	125	0	1	1	0	5	0	2	1	2	37	0	0	1
32	2	100	121	50	89	0	89	0	0	0	1	15	1	2	3	3	50	1	0	0

33	1	10	10	40	87	0	87	0	1	1	1	50	0	2	1	3	45	1	0	1
34	1	60	0	66	61	0	61	0	0	0	0	99	40	2	0	2	51	1	0	1
35	3	80	2	12	35	1	10	1	0	0	1	30	0	1	3	3	42	1	1	1
36	2	60	0	17	110	0	110	0	1	1	0	0	0	2	0	2	37	0	0	0
37	1	100	0	58	32	0	32	0	1	1	0	0	0	1	0	2	50	0	0	0
38	1	40	0	1	100	0	100	0	0	0	0	95	1	2	0	3	55	0	0	1
39	2	70	0	1	119	0	119	0	1	1	0	5	0	1	0	2	42	0	0	0
40	2	80	9	14	110	0	110	0	1	1	0	50	1	2	2	3	45	1	0	0
41	1	70	13	70	143	0	143	0	1	1	0	0	0	2	1	2	50	1	0	0
42	1	40	3	31	82	0	82	0	1	0	1	80	5	1	2	3	47	1	0	1
43	1	10	0	21	75	0	75	1	1	1	0	0	0	1	1	2	60	0	0	0
44	1	90	1	47	12	0	12	0	0	0	1	5	0	3	2	3	41	1	0	1
45	1	10	0	17	112	0	112	0	1	0	0	99	0	1	0	2	39	0	0	0
46	2	80	7	39	90	0	90	0	1	1	0	15	1	1	0	2	59	0	0	0
47	1	10	5	50	120	0	120	0	1	1	0	10	0	1	1	2	66	1	0	1
48	3	80	0	3	99	0	99	0	1	1	0	10	0	2	1	2	50	1	0	0
49	1	10	0	49	109	0	109	0	1	1	0	5	0	2	1	2	37	1	1	0
50	2	40	2	24	26	0	26	1	0	0	1	0	0	2	2	3	54	1	1	1
51	4	4	4	40	114	0	114	0	1	1	0	5	0	1	0	2	58	0	0	0
52	3	70	0	5	56	0	56	0	0	0	1	20	0	1	0	1	32	0	0	0
53	1	60	1	4	116	0	116	0	1	0	0	0	0	2	1	2	45	1	0	0
54	2	90	0	5	68	0	68	0	1	1	0	20	1	1	0	2	38	0	0	0
55	2	20	7	28	51	0	51	1	1	1	0	5	0	3	3	3	37	1	0	0
56	1	80	0	23	25	0	25	1	0	0	1	50	0	3	2	3	55	1	0	1
57	1	80	0	13	95	0	95	0	1	0	0	0	0	2	1	3	40	1	0	1
58	2	60	6	1	50	0	50	1	1	1	0	70	0	2	3	3	47	1	0	1
59	2	60	10	64	120	0	120	0	1	1	0	5	0	2	1	2	45	0	0	0
60	3	90	0	6	43	1	34	1	0	0	1	99	0	1	0	3	54	1	0	1
61	3	90	0	71	118	0	3	1	0	0	1	5	0	2	0	3	70	1	0	0
62	3	70	0	9	113	0	96	1	1	1	0	0	0	2	0	3	41	1	0	1
63	1	60	5	23	116	0	116	0	1	1	0	5	1	1	0	2	39	1	0	0
64	1	70	0	0	134	0	134	0	1	1	0	5	0	2	0	2	46	0	0	0
65	4	4	0	32	84	0	84	1	1	1	0	90	0	1	1	3	50	1	0	0
66	3	70	10	40	126	1	50	1	0	0	1	99	0	2	1	3	38	1	0	1
67	2	80	0	29	167	1	70	1	1	1	0	50	0	1	0	2	47	0	1	0

68	1	10	33	46	1	0	1	0	0	0	1	0	0	3	3	3	69	1	0	1
69	1	10	3	60	110	0	110	0	1	1	0	10	0	1	1	2	55	1	1	0
70	2	60	10	40	105	0	105	0	1	1	0	10	1	1	0	2	47	0	0	0
71	1	10	0	46	31	0	31	1	1	0	1	5	0	3	2	2	40	1	0	0
72	4	4	0	10	110	0	110	0	1	1	0	0	1	1	0	2	36	0	0	0
73	1	40	0	65	111	0	111	0	1	1	1	5	0	2	1	2	43	0	0	0
74	1	60	5	13	75	0	75	0	1	1	1	10	0	2	3	3	65	1	0	1
75	1	60	10	59	110	0	110	0	1	1	0	99	0	2	3	2	40	0	0	1
76	1	80	10	20	95	0	95	0	1	1	0	5	1	1	0	3	49	1	0	0
77	2	60	1	25	110	0	110	0	0	1	0	99	0	2	3	3	53	1	0	1
78	0	0	0	15	107	0	107	0	0	0	1	90	0	2	0	2	61	0	0	1
79	1	10	10	71	62	0	62	0	1	1	0	50	0	1	0	2	57	0	0	0
80	1	90	16	41	102	0	102	0	1	0	0	0	0	2	0	3	44	0	0	1
81	2	90	13	12	101	0	40	1	1	1	1	99	0	2	1	2	62	1	0	0
82	1	70	0	56	133	0	133	0	0	0	1	0	1	2	1	3	50	1	0	1
83	2	80	0	0	132	0	132	0	1	1	0	40	0	2	0	2	47	0	0	1
84	1	90	2	19	128	0	128	0	1	1	0	5	14	2	1	2	42	1	0	1
85	3	90	5	0	18	1	13	1	0	0	1	99	5	1	0	2	43	0	0	0
86	1	10	12	51	100	0	100	0	0	0	0	0	0	2	0	1	56	1	0	0
87	1	30	0	41	14	0	14	1	1	1	0	5	0	2	3	2	40	1	1	1
88	4	4	31	15	107	0	107	0	1	1	0	30	5	1	2	1	53	1	0	0
89	4	4	11	67	103	0	103	0	1	1	0	0	4	1	0	1	41	0	0	0
90	1	10	0	15	10	0	10	1	1	1	0	5	0	2	3	2	51	1	0	0
91	2	90	0	16	94	0	94	0	1	1	0	5	1	1	1	2	47	1	0	0
92	4	4	10	55	107	0	107	0	1	1	0	10	0	2	2	2	43	1	0	0
93	1	60	0	15	107	0	107	0	1	1	0	5	0	1	3	2	41	1	0	1
94	2	60	20	15	110	0	110	0	0	0	0	0	5	2	2	3	36	1	0	1
95	2	90	0	79	75	0	75	0	1	1	0	10	0	2	1	1	49	0	0	0
96	2	60	10	21	60	0	60	0	1	1	1	80	1	1	1	2	60	1	0	0
97	3	70	0	77	130	0	130	0	1	0	0	99	2	2	0	2	49	0	0	1
98	2	70	15	22	129	0	129	0	1	1	0	10	14	1	3	2	38	1	1	0
99	4	4	10	34	129	0	129	0	1	1	0	80	1	1	2	1	36	1	0	0
100	0	0	0	94	90	0	90	1	1	1	0	0	1	2	3	2	48	1	1	0
101	1	70	0	67	121	0	121	0	1	1	0	5	1	2	1	2	41	0	1	1
102	1	60	8	30	56	0	56	1	1	1	1	80	30	1	2	3	51	1	0	1

103	1	20	33	132	92	0	92	0	1	1	0	99	10	2	0	3	58	0	0	1
104	2	30	10	30	97	0	97	0	1	1	1	5	5	2	0	3	52	0	0	1
105	4	4	10	19	92	0	92	0	1	1	1	50	5	3	1	3	56	1	0	1
106	4	4	20	48	87	0	87	1	0	0	1	0	0	2	2	2	66	1	0	1
107	0	0	10	31	26	0	26	0	1	1	0	5	0	2	3	3	30	1	0	1
108	2	90	2	43	101	0	101	0	1	1	0	5	0	2	1	2	44	0	0	0
109	1	90	10	52	101	0	101	0	1	1	0	5	0	1	1	1	39	1	0	0
110	1	90	10	15	101	0	101	0	1	1	0	5	1	3	0	2	50	0	0	1
111	4	4	10	13	101	0	101	0	1	0	1	95	0	1	0	2	48	0	0	0
112	1	30	8	26	66	0	66	0	1	1	0	5	5	2	2	1	38	0	0	0
113	2	100	1	51	100	0	100	0	0	1	1	0	0	2	0	2	51	0	0	1
114	1	90	32	74	100	0	100	0	0	0	0	5	0	2	0	2	67	0	0	0
115	0	0	0	36	62	0	62	0	0	0	0	99	0	2	0	2	42	0	0	1
116	4	4	0	20	45	1	24	1	1	0	0	5	5	2	3	3	41	1	1	1
117	4	4	10	70	100	0	100	0	1	1	0	5	0	1	0	1	45	0	0	0
118	1	60	1	24	100	0	100	0	1	0	0	5	5	1	0	1	35	0	0	1
119	4	4	10	68	110	0	110	0	1	1	0	5	0	2	3	2	35	1	0	0
120	0	0	10	60	99	0	99	0	1	1	0	0	0	2	2	2	47	0	0	1
121	1	80	10	59	49	1	43	1	1	1	0	0	0	2	0	2	28	0	1	1
122	2	60	10	44	99	0	99	0	1	1	0	10	1	1	0	2	48	0	0	0
123	4	4	10	30	32	1	28	1	1	0	0	5	0	1	3	2	42	0	1	1
124	1	90	10	15	98	0	98	0	1	1	0	5	5	2	0	1	39	0	0	0
125	0	0	0	3	97	0	97	0	0	1	0	55	0	1	0	2	63	0	0	1
126	1	60	2	23	62	0	62	0	1	0	0	0	0	1	0	1	59	0	0	0
127	1	100	0	37	96	0	96	0	1	1	0	5	15	2	1	1	49	0	0	0
128	1	60	5	40	95	0	95	0	1	0	0	5	0	1	0	1	50	1	0	0
129	1	90	0	1	94	0	94	0	0	0	0	0	50	2	0	2	51	1	0	0
130	2	100	3	38	127	1	24	1	0	1	0	35	5	1	0	2	79	0	0	1
131	0	0	0	2	120	1	15	1	1	1	0	99	0	2	1	2	52	1	1	1
132	2	90	0	10	94	0	94	0	1	0	0	5	0	2	1	2	66	1	0	0
133	1	10	10	70	94	0	94	0	1	1	0	5	0	2	0	2	40	0	0	0
134	1	60	27	3	64	0	64	0	1	1	0	0	20	1	0	2	25	0	0	1
135	4	4	0	11	93	0	93	0	1	1	0	5	4	2	1	2	58	0	0	0
136	4	4	0	27	93	0	93	0	1	1	1	0	0	1	0	2	52	1	0	0
137	3	100	0	49	94	0	94	0	0	0	0	99	0	1	0	2	46	0	0	0

138	3	90	0	10	100	0	100	0	1	1	0	5	0	2	1	2	69	1	1	1
139	3	90	10	0	110	0	110	0	0	0	1	0	10	1	1	3	41	1	1	1
140	0	0	20	109	120	0	120	0	0	0	1	0	2	2	0	3	47	1	1	1
141	4	4	10	28	90	0	90	0	0	0	0	5	50	1	1	3	40	0	0	1
142	2	60	0	3	28	1	20	1	1	1	1	70	5	3	3	3	48	1	1	1
143	2	70	0	6	40	1	34	1	1	1	0	90	4	1	1	3	35	1	1	0
144	1	30	3	41	89	0	89	0	1	1	0	0	0	2	0	2	59	0	0	0
145	1	100	10	61	66	1	80	1	0	0	0	20	15	1	1	3	65	1	1	0
146	1	10	10	25	46	0	46	1	1	1	0	5	0	1	0	2	46	0	0	1
147	1	60	20	60	89	0	89	0	1	1	0	5	0	1	0	2	26	0	0	0
148	4	4	0	13	88	0	88	0	1	1	0	10	70	1	1	2	71	0	1	0
149	1	10	0	11	89	0	89	0	0	0	1	0	5	2	0	3	56	0	0	1
150	0	0	30	55	88	0	88	0	0	0	0	0	1	2	0	3	38	0	0	1
151	4	4	40	121	18	1	5	1	0	0	0	20	0	3	3	3	59	1	1	1
152	1	50	2	25	15	1	6	1	1	1	1	5	0	2	3	3	44	1	1	1
153	2	100	0	19	10	1	5	1	1	0	1	99	20	2	0	3	63	0	0	1
154	1	60	20	38	48	0	48	0	1	0	0	5	5	3	0	1	53	1	1	1
155	4	4	20	15	107	0	107	0	1	1	0	0	0	1	0	2	43	0	0	1
156	1	5	20	11	97	0	97	0	1	1	0	5	0	2	3	2	41	1	1	0
157	0	0	20	43	51	0	51	0	1	1	0	0	0	2	1	2	64	1	1	0
158	4	4	2	44	106	0	106	0	0	1	0	0	0	1	1	2	42	0	0	0
159	1	30	10	2	57	1	32	1	0	0	0	5	0	1	2	2	51	1	1	1
160	2	70	18	9	121	0	121	0	1	1	1	80	5	1	0	3	47	0	0	0
161	1	10	50	104	121	0	121	0	1	1	0	0	0	2	1	2	41	1	0	1
162	3	70	0	0	36	1	29	1	0	0	1	99	0	2	1	3	45	1	1	1
163	1	90	10	8	12	1	12	1	0	0	0	40	15	2	0	3	33	0	0	1
164	1	60	20	39	86	0	86	0	1	1	1	5	5	1	1	3	45	1	0	1
165	1	40	10	22	42	0	42	0	1	1	0	5	14	2	1	3	44	0	0	1
166	2	90	0	51	84	0	84	0	1	1	0	0	0	2	1	2	71	0	0	0
167	1	10	10	9	14	0	14	0	0	0	0	40	1	2	3	3	57	1	1	1
168	1	40	10	22	49	0	49	0	0	0	1	95	0	1	1	3	63	0	0	1
169	2	90	0	0	83	0	83	0	0	0	1	99	0	3	1	3	47	0	0	1
170	0	0	31	65	84	0	84	0	1	1	0		0	1	1	1	47	0	0	1
171	3	100	2	41	19	1	7	1	0	0	1	70	5	2	3	3	43	1	0	1
172	0	0	25	42	59	0	59	0	1	0	0	0	4	1	0	2	59	0	1	0

173	3	100	0	0	120	0	120	0	0	0	1	99	0	1	0	3	55	1	0	0
174	1	90	0	7	108	0	108	0	1	1	1	5	1	2	0	2	41	0	0	0
175	4	4	0	11	83	0	83	0	1	1	0		4	1	1	1	51	1	0	0
176	3	70	0	98	24	1	10	1	1	0	1	0	1	3	3	2	44	0	0	0
177	3	100	0	37	60	0	60	0	1	0	0	10	1	2	0	3	74	0	0	0
178	2	90	0	0	120	0	120	0	1	1	1	0	1	2	1	2	57	0	0	0
179	3	90	0	0	66	1	55	1	0	0	1	0	5	2	1	3	38	1	0	1
180	2	70	3	5	81	0	81	0	0	0	0	40	0	2	0	1	63	0	0	0
181	2	90	0	0	81	0	81	0	1	0	0	0	0	2	1	1	51	0	0	0
182	3	100	0	0	81	0	81	0	1	1	0	30	0	2	2	3	39	0	0	0
183	4	4	5	19	54	0	54	0	1	0	0		0	2	1	2	60	0	0	0
184	1	60	2	72	81	0	81	0	1	0	1	10	5	1	1	1	53	0	0	1
185	0	0	10	10	27	0	27	0	1	0	0	0	0	1	0	2	55	0	0	0
186	0	0	5	10	49	0	49	0	1	1	0	5	0	2	0	2	42	1	0	0
187	2	60	20	61	56	1	50	1	1	1	0	10	0	2	3	3	40	1	1	1
188	3	70	0	3	24	1	12	1	0	0	1	99	20	2	0	2	47	0	0	1
189	1	90	30	70	79	0	79	0	1	1	1	40	0	3	2	2	55	0	0	0
190	1	70	30	30	99	0	53	1	1	1	0	99	20	2	0	3	32	0	0	0
191	2	90	0	0	99	0	99	0	0	0	0	5	40	2	1	3	46	1	1	0
192	1	40	10	23	98	0	98	0	1	1	0	0	0	2	1	2	62	1	0	0
193	1	80	30	79	97	0	97	0	1	1	0	0	0	1	2	1	43	0	0	1
194	0	0	0	7	97	0	97	0	1	1	0	5	5	1	1	1	48	0	0	0
195	1	60	5	20	97	0	97	0	0	0	0	0	0	2	0	3	45	1	0	0
196	1	60	8	3	97	0	97	0	0	0	0		10	2	0	3	43	1	0	1
197	4	4	5	6	55	1	40	1	1	1	0	50	4	1	1	2	55	0	0	0
198	1	40	0	0	70	1	66	1	1	0	1	95	5	2	0	2	55	1	0	0
199	1	60	32	65	76	0	76	0	1	1	0	5	0	2	1	2	46	0	0	0
200	3	90	0	21	76	0	76	0	0	0	0	0	4	1	2	3	51	0	0	0
201	1	60	0	12	76	0	76	0	1	0	0	10	0	2	0	2	61	0	0	0
202	1	60	23	55	76	0	76	0	1	0	0	5	30	2	0	3	51	0	0	0
203	0	0	0	0	75	0	75	0	1	1	0	5	0	1	0	1	45	0	0	0
204	1	70	0	3	75	0	75	0	1	1	1	99	10	2	0	3	63	0	0	0
205	1	80	0	6	75	0	75	0	1	1	1	0	0	2	0	2	52	0	0	0
206	4	4	7	14	75	0	75	0	1	1	1	0	0	2	0	1	46	0	0	0
207	4	4	10	30	70	1	58	1	1	1	0	30	20	2	2	3	71	1	1	0

208	0	0	6	14	12	0	12	0	1	1	0	30	20	2	2	3	41	1	0	1
209	0	0	0	0	26	0	26	0	1	1	0	5	0	2	0	1	30	1	0	1
210	4	4	0	10	27	0	27	0	1	1	0	0	0	1	0	3	36	1	0	1
211	1	70	10	39	10	1	10	1	1	1	0	5	0	2	1	2	35	1	0	0
212	2	90	0	13	92	0	92	0	1	0	0	50	10	2	2	2	69	1	0	0
213	1	60	0	2	10	0	10	0	1	1	0	5	1	1	0	1	45	1	0	0
214	4	4	0	14	4	1	4	1	0	0	0		30	1	0	3	34	0	0	1
215	1	70	0	11	10	0	10	0	0	0	0	0	5	2	1	3	61	1	0	1
216	1	90	0	7	29	0	8	1	0	0	0	80	1	2	1	3	58	1	0	1
217	1	40	14	27	91	0	91	0	1	1	0	5	0	1	0	2	54	0	1	0
218	0	0	0	0	91	0	91	0	1	1	0	5	1	1	0	1	62	0	0	0
219	0	0	0	18	11	1	10	1	0	0	0	99	0	3	3	3	48	1	1	0
220	4	4	10	45	44	0	44	0	0	0	0		0	2	0	3	44	0	0	0
221	1	60	5	8	120	0	120	0	1	1	0	5	5	1	0	1	39	0	0	0
222	1	60	2	2	19	1	14	1	0	0	1	5	1	1	0	3	52	1	0	0
223	1	60	0	0	84	0	84	0	0	0	1	99	1	1	0	3	37	0	0	0
224	0	0	10	15	15	0	15	0	1	1	0	10	0	1	1	1	49	0	0	0
225	1	10	20	32	120	0	120	0	1	1	1	5	1	1	1	1	43	1	0	0
226	4	4	30	70	28	0	28	0	1	1	0	5	0	2	3	2	43	1	0	0
